# Preparation and Properties of High Solid Content and Low Viscosity Waterborne Polyurethane—Acrylate Emulsion with a Reactive Emulsifier

**DOI:** 10.3390/polym10020154

**Published:** 2018-02-06

**Authors:** Zhewen Zhu, Ruiqi Li, Chaoying Zhang, Shuling Gong

**Affiliations:** College of Chemistry and Molecular Sciences, Wuhan University, Wuhan 430072, China; zhuzhewen@whu.edu.cn (Z.Z.); lirq@whu.edu.cn (R.L.); whu_chem_zcy@163.com (C.Z.)

**Keywords:** coatings, polyurethane-acrylate, reactive emulsifier, emulsion polymerization

## Abstract

High solid content waterborne polyurethane-acrylate (WPUA) emulsions have been successfully synthesized in two steps. Firstly, we prepared a waterborne polyurethane emulsion, then reacted it with acrylate monomer by emulsion polymerization using the semi-continuous seeded method. The effects of the type and amount of emulsifier, the amount of dimethylolpropionic acid (DMPA), the choice of capping group, the ratio of PU/PA, and the method of adding a water-soluble monomer to the properties of the composite emulsion were investigated. The reactive emulsifier replaced the traditional emulsifier and there were no metal ions introduced to the reaction, whether by the emulsifier or the initiator. Through a variety of tests, we proved that the prepared emulsion has the advantages of small particle size, narrow distribution, good stability, good performance of the film, and solid content of 46%.

## 1. Introduction

Before the advent of water-based coatings, solvent-based paints have evolved considerably to achieve industrial production and are widely used in many areas of human life. Regarding solvent-based coatings, the main solvents are butanone, cyclohexanone, ethyl acetate, and toluene. These organic solvents tend to be highly volatile and toxic, flammable and explosive, which is not only dangerous, but also a threat to the environment and human health [1,2,3]. With the popular concept of environmental protection, a large number of relevant environmental laws and regulations have been formulated to control the emissions of volatile organic compounds (VOC). These measures limit the application of solvent-based coatings, but also promote the development of water-based paint. The difference between water-based coatings and solvent-based coatings is that the former uses water as a dispersion medium, it is safe, environmentally friend, etc. In industrial production, water-based coatings can be prepared by controlling the reaction conditions and optimizing the production process, which is consistent with the properties of solvent-based coatings and has a similar morphology [4,5,6].

Due to its special structure, waterborne polyurethane has unique physical and chemical properties. For example, hardness can be adjusted, as can low temperature resistance, good wear resistance, good adhesion, and so on. Waterborne polyurethane coatings with low VOC emissions and good product performance have drawn wide public concern [7,8]. However, due to the limitations of the molecular structure of waterborne polyurethane, the film has some disadvantages, such as heat resistance, water resistance, solvent resistance, and poor gloss [9,10]. Polyacrylate (PA) has the advantages of good water resistance, aging resistance, good gloss, and good mechanical properties. In view of this, PU and PA can be combined by physical blending or chemical copolymerization to obtain a waterborne polyurethane-acrylate emulsion (WPUA), fully reflecting their respective advantages [11,12].

Sultan et al. [13] terminated the PU segment by hydroxyethyl methacrylate (HEMA), then reacted it with acrylic monomer. The solid content of PUA copolymers decreases gradually from higher PU/AC ratios to lower ones. The highest solid content is 38.15% and the lowest is 28.6%. The polyurethane-acrylic emulsion exhibited synergistic effects between the two polymers and revealed a remarkable improvement in numerous coating properties. Sebenik et al. [14] treated WPU latex particles with amphiphilic structures as polymeric surfactant acrylic monomers were added into the WPU emulsion, then entered into the WPU latex particles, adding and reacting to form core-shell structures composed of a polyurethane-acrylic emulsion, and found that the polymerization in the presence of the PU dispersion showed an intermediate behavior between Smith-Ewart cases I and II. The solid content of the emulsion they prepared was about 34%. However, for use as a coating, the solid content of the emulsion was insufficient. Kim et al. [15] used sodium dodecyl sulfonate (SDS), as the emulsifier to prepare WPUA, and found that the grafting rate of methyl methacrylate (MMA) was increased with the increase of the hard segment content of the waterborne polyurethane emulsion in the waterborne polyurethane acrylate emulsion prepared by seed emulsion polymerization. They found that the average particle size after the seeded emulsion polymerization and grafting efficiency increased as the hard segment contents increased. Li et al. [16] synthesized a high solid content WPUA by the monomer dilution method and the emulsification method, respectively. They found that the DMPA and emulsifier content increase brings about smaller particles and better storage stability of the emulsions, but the water resistance decreases. The emulsion used SDS as the emulsifier and could be stable. However, when the emulsion became a film, there was some shrinkage.

Over the past years, the research of waterborne polyurethane-acrylate emulsion has seen rapid development, but there are still some unsolved problems. Since the emulsion we prepared is used as the coating of cars, the film should be smooth enough and have a particular luster. When SDS, or other which contains any metal ions, is used as an emulsifier, it is physically adsorbed onto the surface of the latex particles. During the drying process of the emulsion, the residual small molecule emulsifier migrates to the surface, resulting in shrinkage, a decrease of the coating gloss, and other performance. Additionally, if the emulsion solids content is low, this would lead to a longer film-forming time and low production efficiency. However, the viscosity of the emulsion increases as the solid content increases. This is not conducive to actual production.

In this paper, we need to solve the problem of residual emulsifier and retain the low viscosity and high solid content of the emulsion. Therefore, we used the amphiphilic polyurethane macromolecules, supplemented by a small amount of reactive emulsifier SE-10 instead of the traditional ion/non-ionic emulsifier on the acrylic monomer pre-emulsification. SE-10, through covalent bond polymerization, is bonded to the molecular chain of the macromolecular polymer. As a result it cannot be displaced from the interface as easily as traditional surfactants, which are only physically bonded [17,18,19,20,21,22]. Permanent attachment to the dispersed phase also have environmental and economic advantages. This results in lower use of surfactants and smaller contamination of sewage [23]. Reactive surfactants can be used to obtain redispersable lattices, latexes with functionalized surfaces, or dispersions with increased stability [24,25].

We determined the polymerization reaction conditions and the synthesis process, and discuss the different dosages of emulsifier and hydrophilic monomer DMPA on the properties of emulsion polymerization and emulsion stability. By changing the ratio of PU/PA, we have also synthesized composite emulsions with different properties. The results of the tests show that the prepared emulsion has excellent properties and has the potential to replace solvent-based coatings.

## 2. Materials and Methods

### 2.1. Materials

Acylic acid (AA), methacrylic acid (MAA), butyl acrylate (BA), *n*-butyl methacrylate (BMA), styrene (St), hydroxyethyl methacrylate (HEMA), 1-dodecanethiol, ammonium persulfate (APS), trimethylamine (TEA), ammoniumhydroxide (NH_3_∙H_2_O), sodium dodecyl sulfonate (SDS), dodecanethiol, *N*,*N*-dimethylethanolamine (DMEA), 1,4-Butylene glycol (BDO), butanedioic acid and Alkylphenolpolyoxyethylene ether (OP-10) were provided by Sinopharm Chemical Reagent Co., Ltd. (SCRC, Shanghai, China). Reactive emulsifier SE-10 was purchased from Foshan Kodi Gas Chemical Industry Co., Ltd (Foshan, China). Isophoronediisocyanate (IPDI), polycarbonate diol (PCDL, the type of polycarbonate diol was 1,5-pentanediol and 1,6-hexanediol) were provided by Zhongshan Daoqum Co., Ltd. (Zhongshan, China). Dimethylolpropionic acid (DMPA) was purchased from Shanghai Aladdin Co., Ltd. (Shanghai, China). Dibutyltindilaurate (DBTDL) was purchased from Beijing Sananhua Co., Ltd. (Beijing, China). In order to eliminate the inhibitor, the activated carbon adsorption method and alkali washing with NaOH or NaHCO_3_ were used for the water-soluble monomer (AA, MAA, HEMA) and other monomer (BA, BMA, St), respectively. Other materials were used without further purification.

### 2.2. Methods

#### 2.2.1. Preparation of the Waterborne Polyurethane Emulsion

An appropriate amount of PCDL was added into three dried flasks, the temperature was increased to 110 °C, and the water reduced for 1 h. Cooling to 85–90 °C, IPDI, DPMA, and DBTDL were added to react for 1 h. (–C=C ended PU pre-polymer), cooled to 60 °C, and the amount of HEMA was added into the flasks to react all –NCO for 3 h. (–OH/–COOH ended PU prepolymer). This was cooled to 60 °C, and half the amount of HEMA was added into the flasks to react one end –NCO of PU for 3 h. Then, BDO/butanedioic acid was added into the flasks to end cap the other end for 3 h. The pre-polymer was cooled to 40 °C and added with a proper amount of TEA to neutralize for 0.5 h. The waterborne polyurethane emulsion (WPU) was obtained by adding deionized water and dispersed in water under high-speed stirring.

#### 2.2.2. Preparation of Waterborne Polyurethane—Acrylate Composite Emulsion

AA, MAA, BA, BMA, HEMA, St, and dodecanethiol were added into the three-necked flask to mix with WPU. After mixing, the temperature was raised to 80 °C, the emulsifier and water was mixed for pre-emulsification for 0.5 h, with ammonia to adjust the pH = 8. After that, 80% of the pre-emulsion was removed to the constant pressure dropping funnel, 20 wt % of the APS was dissolved in water and added to the flask, and the remaining 80% pre-emulsion and initiator were slowly added dropwise together. The reaction was continued for 4 h after completion of the dropwise addition. During the reaction, the amount of initiator was added according to the degree of reaction of the monomer and the viscosity of the resin, and the temperature could be raised to 90 °C at the later stage of the polymerization. After that, the mixture was cooled to 60 °C, and the neutralizing agent DMEA was added to obtain an aqueous polyurethane-acrylate composite emulsion.

**Figure 1:**
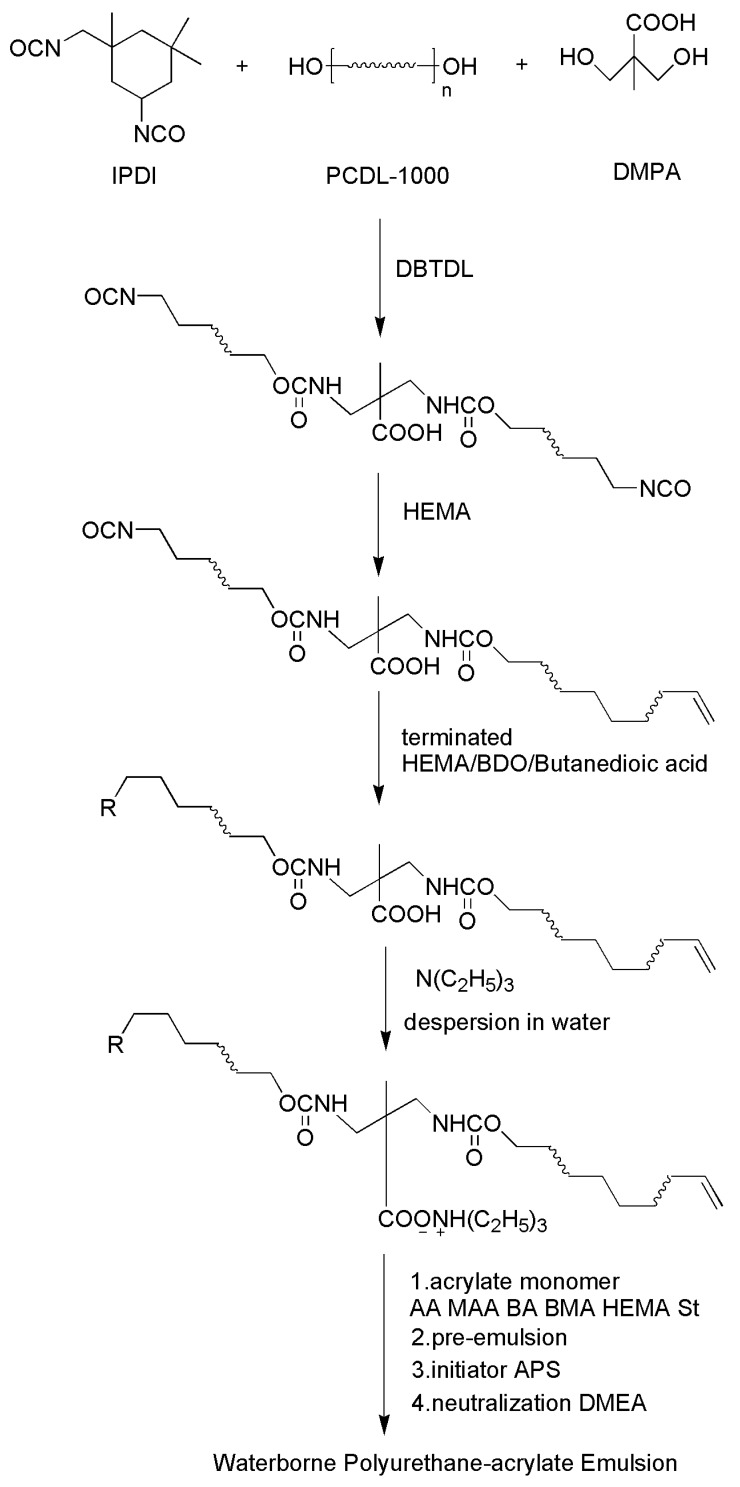
Synthetic route of WPUA emulsion. R was –C=C/–OH/–COOH.

The ratio of NCO/OH in the synthesis of the PU pre-polymer was 1.5/1. The neutralization degree of the whole reaction was 0.9. The ratio of AA/MAA/BA/BMA/HEMA/St in the synthesis of the WPUA emulsion was 1.2/2.6/10.9/40.8/20/24.5. The content of 1-dodecanethiol and the initiator are 3% and 2%, respectively. The reaction process shown in Figure 1.

### 2.3. Characterizations

#### 2.3.1. Solid Content

The determination of the solid content is as follows:

(1) $$\mathrm{Solid}\%=\left[\frac{W_{f}}{W_{s}}\right]$$

where *W_f_* and *W_s_* are the weights of dried emulsion and emulsion, respectively.

#### 2.3.2. Coagulum Content

The filterable solids were dried. The coagulum content was then calculated according to Equation (2):

(2) $$\mathrm{Coagulation}\%=\left[\frac{M_{f}}{solid\%\times M_{s}+M_{f}}\right]$$

where *M_f_* and *M_s_* are the weights of dried filterable solids and filtered latex, respectively.

#### 2.3.3. Viscosity

Viscosity was measured with a DV-79 digital viscometer (Worner Lab., Shanghai, China), employing E (10–100 mPa∙s) or F (100–1000 mPa∙s) rotor rotating at a velocity of 750 rpm at 25 °C.

#### 2.3.4. Particle Size

A small amount of emulsion was diluted to 1% with distilled water, and the particle size and particle size distribution of emulsion were determined by using Zetasizer Nano ZS laser particle sizer (Malvern Instruments Ltd., Shanghai, China) at 25 °C.

#### 2.3.5. Transmission Electron Microscopy

Micrographs of the polyurethane–acrylate latex nanoparticles were obtained by JEM-2100 (JEOL Ltd., Tokyo, Japan) transmission electron microscope at an acceleration voltage of 200 kV. The emulsion was thinned to the appropriate concentration. Samples were stained with 3% phosphotungstic acid (PTA) solution. 

#### 2.3.6. Fourier Transform Infrared Spectroscopy

The waterborne emulsion was dropped on the potassium bromide tablets and dried under an infrared lamp, then put into a Nicolet-Nexus 670 FTIR (Thermo Fisher, Waltham, MA, USA) to analyze in the range from 4000 to 400 cm^−1^.

#### 2.3.7. Stability of the Emulsion

The emulsion was stored at room temperature for six months, or added to a CaCl_2_ solution or diluted with distilled water, and then observed whether there was a phenomenon of stratification, precipitation, flocculation, and so on, to evaluate its storage stability, chemical stability, and dilution stability, respectively.

#### 2.3.8. Gel Permeation Chromatography

The number and average molecular weights were determined by gel permeation chromatography with tetrahydrofuran (THF) as the eluent, and polystyrene standards, as calibrated.

#### 2.3.9. Preparation of Films

The emulsion was poured onto polytetrafluoroethylene plate and dried for 24 h at room temperature, then dried at 60 °C for 48 h to obtain the film.

#### 2.3.10. Water Resistance of the WPUA Films

The film was cut to a size of 3 cm × 3 cm and weighed accurately at room temperature. The sample was then immersed in deionized water for 24 h. After removing the water on the surface of the adhesive film, the quality of the wet film can be quickly weighed. The water absorption rate of the adhesive film can be calculated by substituting the measurement results into the formula:

(3) $$\omega \%=\left[\frac{W_{1}-W_{0}}{W_{0}}\right]$$

where *W*_0_ and *W*_1_ are the weights of the film before and after absorbing water, respectively.

#### 2.3.11. Solvent Resistance of the WPUA Films

The film was cut to a size of 3 cm × 3 cm. The samples were then immersed in tetrahydrofuran (THF) for 24 h. The state of the sample after soaking was observed.

#### 2.3.12. Differential Scanning Calorimetry Measurements

The glass transition temperature (*T*_g_) of the polymer was measured in the range from −100 to 200 °C with a Mettler 822e differential scanning calorimeter under a nitrogen atmosphere at a heating rate of 10 °C/min.

#### 2.3.13. Thermogravimetric Analyzer

The heat resistance of the samples was tested on a Setsys16 thermogravimetric analyzer from Setrama, France. The sample weighed about 5 mg, in a nitrogen atmosphere, the heating rate was 10 °C/min, with a heating range of 20–600 °C. This method was used to test the thermal stability of the sample.

#### 2.3.14. Hardness of the WPUA Films

The prepared film was placed on a horizontal surface. The angle of a pencil and adhesive film was about 45 degree and, placed under constant speed force (with the pencil not broken), the pencil was pushed forward to create a 1 cm scratch on the film. According to the pencil from the hardest cartridge to the softest cartridge, the same pencil hardness level is scratched for five traces. When the five pencil marks do not scratch the adhesive film, the film is considered to have reached ‘pencil hardness’ [26].

#### 2.3.15. Mechanical Property Test of the WPUA Films

The adhesive film was cut into an 8 cm × 1 cm sheet shape, and the thickness of the film was measured by a CMT6503 electronic universal testing machine (Optomal Hung Measurement & Control Technology Co., Ltd., Shanghai, China). The tensile strength and elongation at break of the film were measured at room temperature with a tensile speed of 100 mm/min. This method was used to measure the elongation at break of the sample.

## 3. Results and Discussion

### 3.1. Effect of DMPA Dosage in PU of WUPA

In order to allow non-hydrophilic polyurethane to be well-dispersed in water, it is necessary to introduce hydrophilic groups into the polyurethane molecular chain. The introduction of hydrophilic groups has a greater impact on the stability of the emulsion. The better hydrophilicity of the molecular chain, the better the dispersity of the emulsion in the water, and the solution is more stable. When the content of the DMPA is too low, the number of hydrophilic groups on the surface of the particles is small, and it is difficult to form a stable emulsion.

As shown in the Table 1, with the increase in the amount of DMPA, the stability of the emulsion improves, and the surface of the film also becomes smoother. This is because the greater the amount of DMPA added, the greater the surface charge density of the particles, and the particles are not easy to agglomerate due to electrostatic interaction. When the amount of DMPA is too low, the hydrophilic groups of PU chains are too few, which cannot disperse stably in water. However, if the amount of DMPA is too large and the hydrophilic group is too large, the water resistance of WPUA will worsen. The viscosity of the emulsion is increased because the system, with respect to the content of –COOH, is increased with the added amount of DMPA. While the emulsion particle size decreases, the particle number increases, and the increase in volume of unit collision probability results in the mutual moving becoming more difficult, thereby increasing the viscosity. Therefore, in order to prepare a stable WPUA emulsion, the amount of DMPA was determined to be 7%.

### 3.2. Effect of PU Terminal Groups on WPUA

Since PU needs graft copolymerization with acrylic acid monomer, one end needs its double bond to be terminated, and the other end can choose double bonds, hydroxyl groups, and carboxyl groups to seal the end. Different end capping has different effects on the properties of the emulsion.

**Figure 2:**
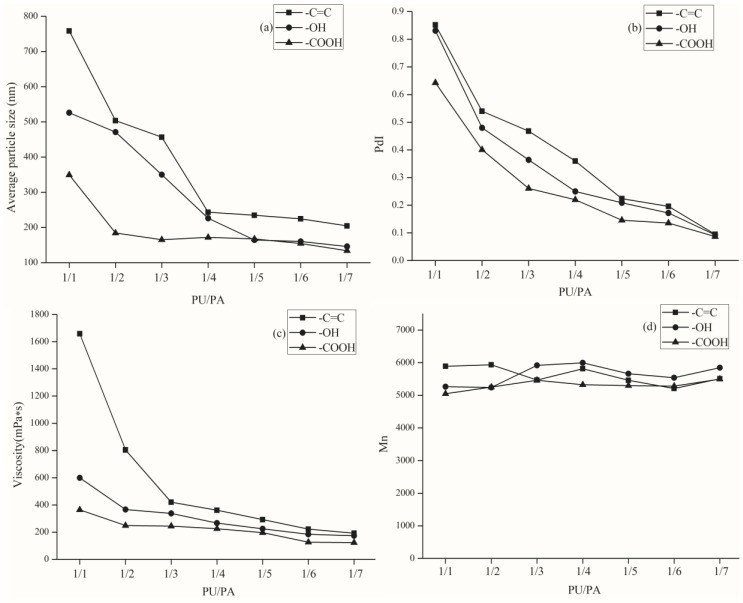
Effect of PU terminal groups on WPUA on (**a**) particle size; (**b**) PdI; (**c**) viscosity; and (**d**) *M*_n_.

From Figure 2a,b, the average particle size of the emulsion end with a double bond is the largest and the distribution is wider. The average particle size of the carboxyl-terminated emulsion is the smallest and the distribution is narrower. This is due to the presence of more double bonds and hydroxyl groups in the –C=C– and –OH–terminated PUs, which are prone to side effects. The carboxyl groups in the –COOH–terminated PU are already neutralized in the preparation of the polyurethane pre-polymer, so there are not a lot of side reactions occurring, and the latex particles are also relatively small in size, and particle size distribution is narrower.

As shown in Figure 2c, because of the existence of two –C=C, chemical cross-linking occurs more easily than with only one –C=C of the PU chains, so the viscosity of the emulsion is greater than the others. The –OH–terminated PU, due to the presence of more hydroxyl groups, also increases the viscosity of the emulsion with strong hydrogen bonding of the acrylic monomer. Especially, with the ratio of PU/PA in the range of 1/1 to 1/4, the difference is more obvious. This is because, in this range, the polyurethane proportion of the total emulsion is more than sufficient. In the range of 1/5 to 1/7, with the reduction of the PU ratio and different ends of the PU, the overall impact of the emulsion is also reduced, whether it is the average particle size, particle size distribution, or viscosity, are not very different.

As shown in Figure 2d, the number-average molecular weight (*M*_n_) of WPUA, no matter what the end group is, is in the range of 5200 to 5900. All of the emulsions are appropriate for high solid content coatings.

Since the carboxyl content of the carboxyl-terminated PU was greater than the PU terminated with the other two groups, the emulsification effect of the PA monomer in the second step reaction was better than that of the other two capped PU after neutralization and salt formation. Considering all aspects, in order to prepare the emulsion with better properties, we chose carboxyl as the end group of PU.

### 3.3. Effect of PU/PA Ratio

In the preparation of the WPUA emulsion, amphiphilic polyurethane macromolecules, in addition to the macromolecule emulsifier, played important roles in the performance of WPUA.

As shown in the Table 2, with the increase of the PU ratio, the water resistance and emulsion stability of WPUA worsens. Since the PU is not easily dispersed in the acrylic monomer during the reaction, resulting in difficulty forming the pre-emulsion, the final emulsion is unstable. Under the same mass, the hydrophilic group provided by PU is greater than that of PA, so the higher the content of PU in the polymerization system, the worse the water resistance of WPUA. After the WPUA emulsion is dried, the carboxyl group will be exposed when the amino acid is volatilized. With infrared light, the carboxyl group will be cross-linked with the hydroxyl group. The WPUA emulsion molecular chain runs through and entangles with each other. The penetration of other molecules becomes difficult, thereby the water resistance and solvent resistance of the film was improved. Since the crosslinked hydroxyl groups all originate from the PA monomer, the water resistance and solvent resistance of the WPUA are both improved when the ratio of PA is increased. The molecular chains of the WPUA emulsions prepared by using acrylic acid as the solvent are interpenetrating and entangled, which makes the permeation of other molecules difficult, thus improving the water resistance and solvent resistance of the film [27]. When the ratio of PU is decreased, the hardness of the WPUA film increases, and the elongation at break decreases. This is due to the special structure of microphase separation in PU, as usually there will be some defects in the crystalline region and amorphous region of the soft segment. After the introduction of PA, the acrylic acid is infiltrated into the polyurethane network to repair the defects, showing a synergistic effect, thus increasing the hardness of the material [28]. On the other hand, with the increase of the ratio of PA, relative movement between the polyurethane and acrylic molecular chains will be inhibited because they are, to a certain extent, intertwined. Thus, the degree of microphase separation is reduced, and the deformation ability of the polymer is weakened, resulting in the decrease of the elongation at break of the film.

**Figure 3:**
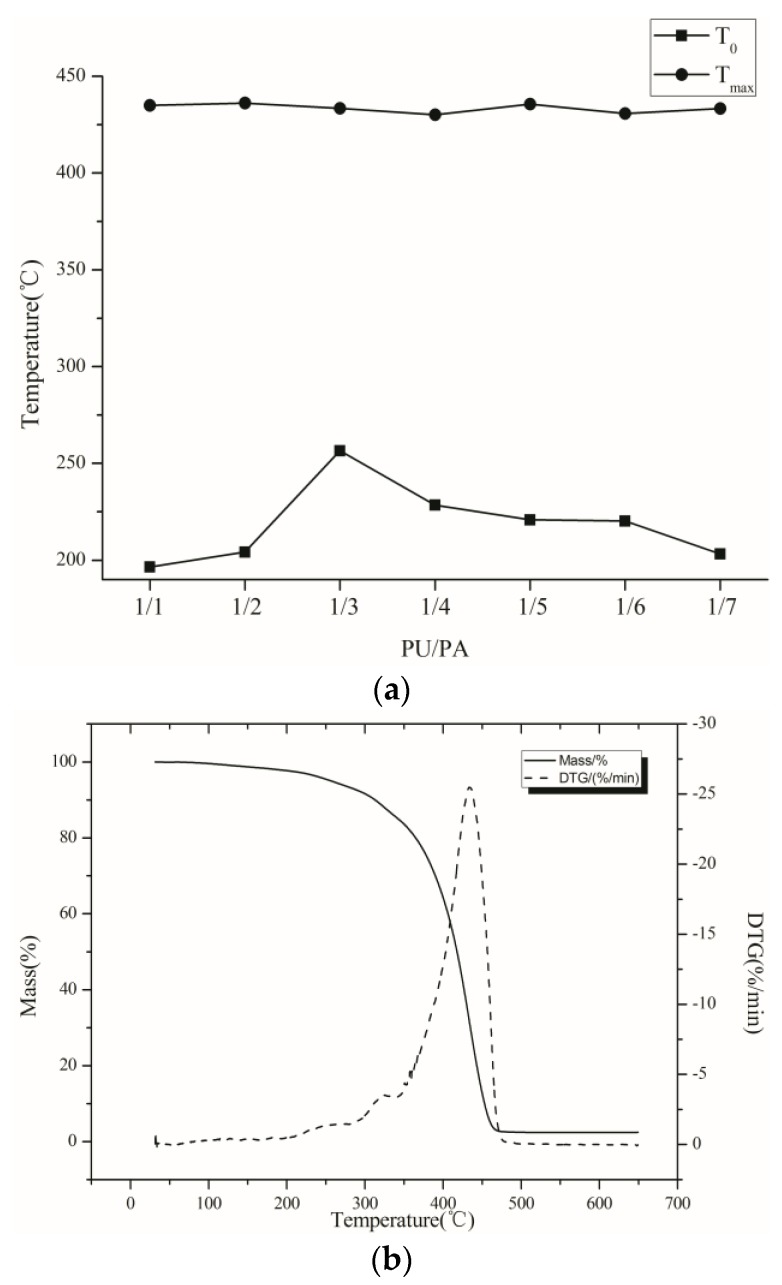
(**a**) Thermogravimetric analysis of different ratios of PU/PA. *T*_0_ is the temperature at which the sample mass decreased 5% and *T*_max_ is the temperature at which the mass loss was the fastest; and (**b**) TGA/DTGA curves when the ratio PU/PA was 1/3.

It is shown from the Figure 3 that with the change of the PU/PA ratio, the maximum weight loss temperature of the film remains nearly unchanged, and the initial weight loss temperature of the film increases firstly, and then decreases with the decrease in PU content. Considering the comprehensive performance of the emulsion, the ratio of PU/PA is defined as 1/3 in this paper.

### 3.4. Effect of Water-Soluble Monomer Addition Method

The stability of emulsion polymerization mainly depends on two aspects: the steric effect of the nonionic water-soluble monomer; and the electrostatic repulsion of the ionic monomers. Therefore, whether the water-soluble monomer can be effectively bonded on the surface of latex particles affects the stability of the emulsion. As shown in the Table 3.

When the MAA/AA is both added in pre-emulsion, the amount of carboxyl groups in the reaction system is relatively small. The carboxyl groups are mostly buried in the latex particles, so that the surface of the latex particles cannot be completely covered with water-soluble monomers to produce sufficient electrostatic repulsion to remain stable, and the latex particles may agglomerate, widening the particle size distribution.

When the MAA/AA is both added to the seed emulsion, the amount of carboxyl groups in the reaction system is relatively excessive. In addition to bonding to the latex surface, extra carboxyl could copolymerize in the aqueous phase, easily producing gel particles. This is makes the residual monomer lack enough carboxyl to react, leading to the new generation of latex particles having poor water solubility, and the stability of the emulsion became worse.

When MAA/AA is added to the pre-emulsion and seed emulsion, respectively, from the table comparing the particle size of the emulsion, it can be seen that the MAA (seed)/AA (pre-emulsion) method has a smaller particle size and a narrower distribution. This is because the mass fraction of MAA (2.6 wt %) is greater than AA (1.2 wt %); the carboxyl of AA is sufficient to cover the surface of the latex particles, and there will be an excess of MAA in the seed emulsion. The hydrophilicity of the latex surface is associated with the water, reducing the amount of free water, thus increasing the viscosity of the emulsion.

In summary, in order to make the hydrophilic monomer distribution better, so as to prepare a more stable emulsion, we the chose the MAA (pre-emulsion)/AA (seed) adding method.

### 3.5. Effect of Emulsifier Content

**Figure 4:**
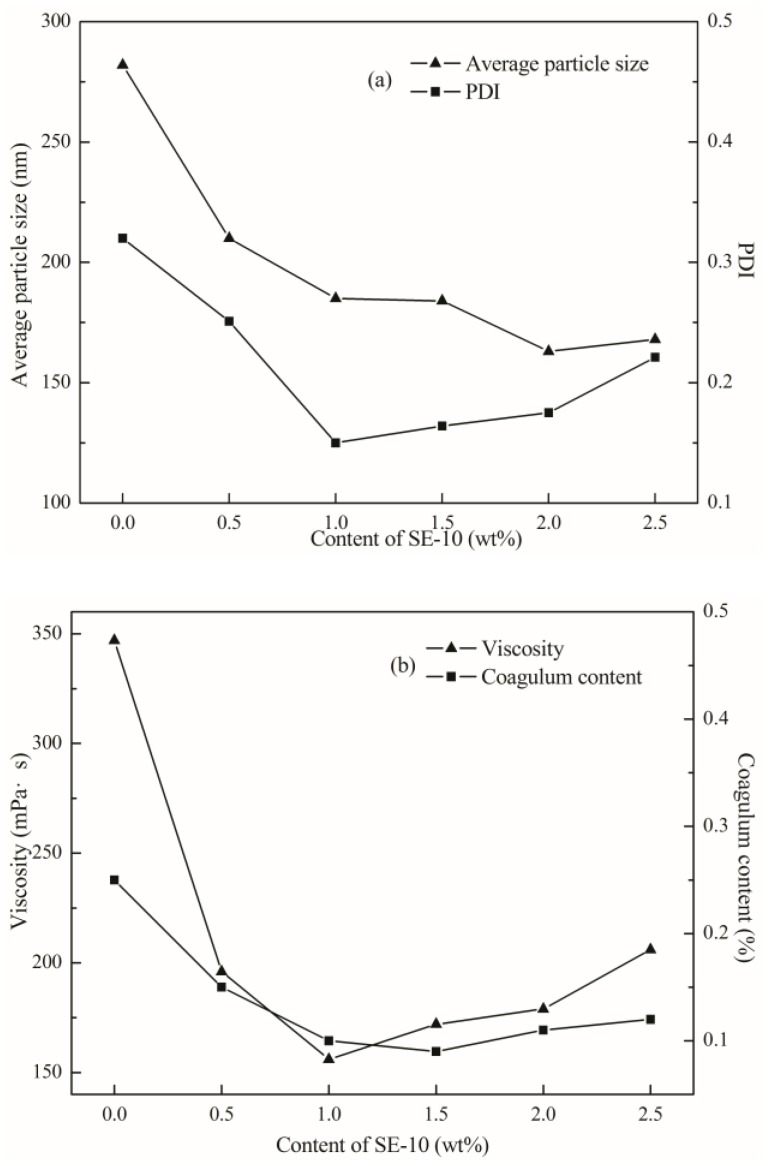
Effect of content of SE-10 on (**a**) particle size and PdI and (**b**) viscosity and coagulum content.

According to the Figure 4a, the particle size of the emulsion decreases with the increase of the emulsifier content. When the amount of SE-10 was less than 1 wt %, the distribution became narrow with the increase of emulsifier amount. When the dosage was 1 to 2.5 wt %, the particle size distribution became broader. As shown in Figure 4b, the viscosity of the emulsion was drastically reduced in the range of 0 to 1% with the addition of the emulsifier and increased in the range of 1 to 2.5 wt %. The coagulum content of the emulsion was significantly decreased in the range of 0 to 1 wt % with the increase of the addition amount, and the change was not significant in the range of 1 to 2.5 wt %.

When the amount of emulsifier is increased, the number of latex particles is increased, as well. Since the average particle size of the polymer became smaller, while the coagulum content was reduced, the stability of emulsion improved. When the amount of emulsifier is too small, the emulsifier molecules cannot cover the surface of the latex particles, and the emulsion coagulum is increased, then it is difficult to maintain stability. The size of the latex particles is not uniform, resulting in the free water having difficulty flowing between the latex particles, so the emulsion viscosity rose [29]. If the amount of emulsifier is too great, it is easy to copolymerize in the water phase to generate the relatively large molecular weight rich hydrophilic group of water-soluble polymer and the adsorption of latex particles, resulting in an uneven latex particle size distribution, and reducing the performance of emulsion [30,31].

Therefore, taking into account the performance of the emulsion, this paper will use a 1 wt % amount of reactive emulsifier.

### 3.6. Comparison of Selection of Emulsifiers

In emulsion polymerization, adding proper amounts of emulsifier can play the role of emulsification and dispersion, and it is important in the stability of emulsion. Different emulsifiers on the performance of the comparison shown in Table 4.

In an ideal emulsion polymerization mechanism, the relationship between the number of latex particles and the emulsifier concentration is Np/[S]^0.6^. The larger [S] is, the larger Np is, and the corresponding latex particle size decreases monotonically with the increase of [S] [32]. Since the molecular weight of SDS (288) and the molecular weight of OP-10 (646) are smaller than the molecular weight of SE-10 (872), the mass concentration of [S] is higher, which leads to the smaller particle size of the prepared SDS. The use of emulsifier SE-10 latex particle size is more uniform, so the gap between the latex particles are more uniform. When sufficient water was used, all emulsion viscosities were also obtained using the SDS/OP-10 emulsion.

The reactive emulsifier SE-10 is chemically bonded to the surface of the latex particles, while the SDS is physically adsorbed on the surface of the latex particles, so it is more likely to migrate. As such, the chemical stability of SE-10 as the emulsifier was better. 

The reactive emulsifier SE-10 molecule contains a double bond which, by covalent bonding with the latex particles rather than by physical adsorption, avoids the resolution or migration of the latex particles. Since the latex particles are subjected to static electricity during the polymerization process, repulsive force, and steric hindrance, the emulsion polymerization stability is improved, and the emulsion performance is improved. Therefore, considering the combination of emulsion and coating performance, the use of reactive emulsifier SE-10 for emulsification is recommended.

### 3.7. FTIR Spectra

**Figure 5:**
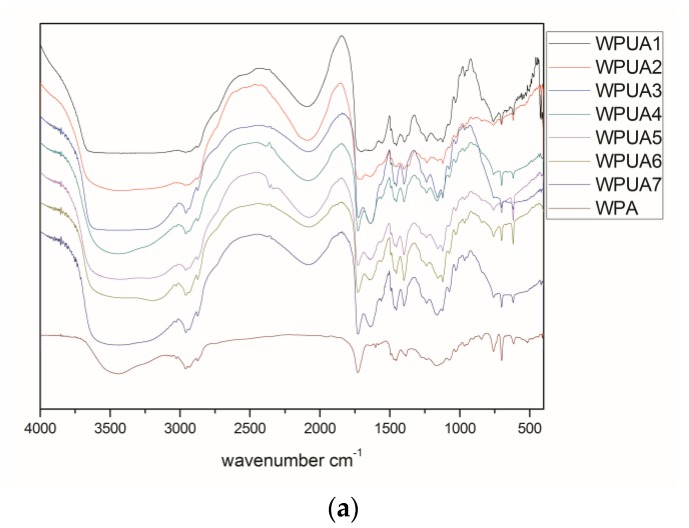

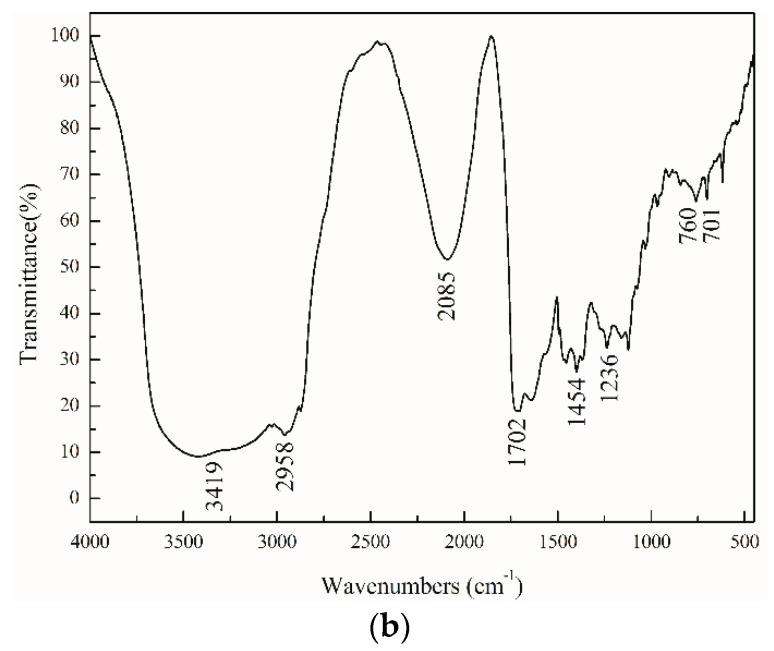
(**a**) FTIR spectra of polyurethane–acrylate that different ratios of PU/PA. In WPUA1 to WPUA7, the ratio of PU/PA = 1/1 to 1/7; and (**b**) FTIR spectra of polyurethane–acrylate with ratio of PU/PA of 1/3.

As shown in the Figure 5, the broad peak at 3419 cm^−1^ is the O–H stretching vibration absorption peak of the hydroxyl functionalized acrylate. The N^+^–H stretching vibration peak is included in the broad peak range from 3800 to 2300 cm^−1^ and in combination with the peak of 2200 to 2000 cm^−1^. The C–H stretching vibration peak is 2958 cm^−1^, while the in-plane bending vibration peak of C–H is 1454 cm^−1^, respectively. The strong absorption peak at 1702 cm^−1^ is the stretching vibration peak of C=O in the urethane and acrylate groups, which moves to a low wave number due to the formation of hydrogen bonds. There is no stretching vibration peak of –NCO at 2274 cm^−1^, indicating that the reaction of the –NCO group in the system is complete. Characteristic absorption peaks of a single substituted benzene ring occur at 760 and 701 cm^−1^. The absorption peak of 1236 cm^−1^ belonging to RO–SO^3−^, show that SE-10 was involved in the polymerization of emulsifier. In addition, the characteristic absorption peak of S–H at 2500–2590 cm^−1^ completely disappeared, indicating that the thiol groups in the chain transfer agent were transferred to the carbon through radical chain transfer. The waterborne hydroxyl functionalized polyurethane acrylate composite emulsion was successfully prepared by emulsion polymerization by infrared analysis.

### 3.8. DSC Analysis

**Figure 6:**
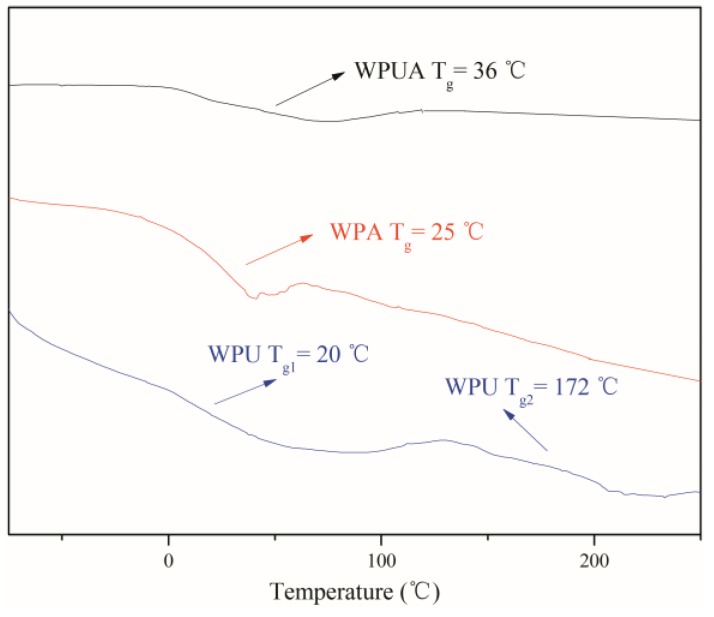
DSC thermogram of WPU, WPA, and WPUA.

As shown in the Figure 6, the *T*_g_ of the composite emulsion WPUA is higher than that of WPA. The compatibility of PU and PA in the WPUA composite emulsion prepared by emulsion polymerization was greatly improved, having an almost homogeneous structure. Thus, in the diagram, WPUA only shows one glass transition temperature [33].

### 3.9. Microstructure of the Latex Particles

**Figure 7:**
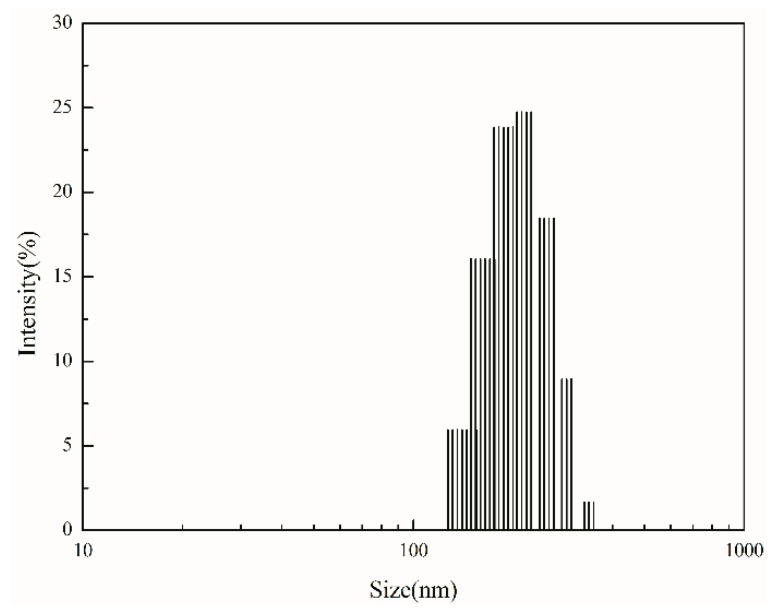
Size distribution of the WPUA latex particles.

**Figure 8:**
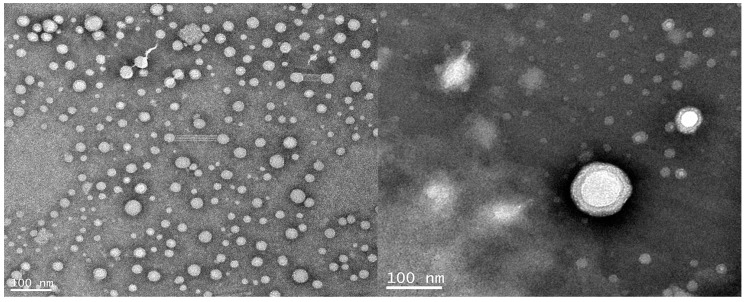
TEM micrograph of the core–shell WPUA latex particles.

The dynamic light scattering method (DLS) was used to determine the particle size distribution of WPUA latex particles, as shown in Figure 7. The average particle size of latex particles was 190 nm, showing a single peak distribution. The microstructure of the latex particles was observed by transmission electron microscopy (Figure 8). The latex particles were approximately spherical with a diameter of about 190 nm and a uniform size, which was consistent with the dynamic light scattering method. As can be seen from the figure, the latex layer is bright, and the outer brightness is weaker, showing the obvious core-shell structure. It is concluded that the core-shell structure of WPUA latex particles can be successfully obtained by seeded emulsion polymerization.

In addition, the “hairy” structure extending from the edge of the core layer of the latex particles to the surface of the shell can be observed from the TEM photos. The reason for this phenomenon may be due to the seed liquid water-soluble monomers tending toward a larger AA distribution of the latex particles on the surface of the core layer in the emulsion copolymerization process, after amination of –COOH ionization for –COO^−^ ions, and the polymer chain segments of the surface of the core layer fully extended into the water phase, and so the TEM micrograph shows the “hairy” structure [34].

## 4. Conclusions

The emulsion of acrylic acid monomer was emulsified with two amphiphilic polyurethane macromolecules and 1% reaction emulsifier SE-10, and the residual emulsifier in the final emulsion was determined. IPDI, PCDL, and DMPA were used in the preparation of the polyurethane chains, respectively, by double bond and carboxyl-terminated on both sides, then polymerized with PA monomer. When the amount of DMPA was 7%, PU/PA was 1/3, and MAA added in the pre-emulsion and AA added in the seed emulsion can be prepared by waterborne polyurethane-acrylic composite emulsion. Through a variety of tests, we proved that the waterborne polyurethane-acrylate core-shell composite emulsion was successfully prepared with small particle size, narrow distribution, good stability, and good adhesive film properties. The solid content of the emulsion prepared by this method could reach 46%. It can be predicted that the coatings prepared with this emulsion with low VOC emission can gradually replace the solvent-based coatings.

## Figures and Tables

**Table 1:** Effect of DMPA dosage on the properties of the emulsion.

*w* (DMPA)/%	9	8	7	6	5
Emulsion stability	Stable	Stable	Stable	Stable	Unstable
film appearance	Smooth	Smooth	Smooth	Unsmooth	Unsmooth
Water absorption (%)	15.4	13.7	10.1	7.9	4.6
Viscosity/mPa∙s	546	412	336	298	267
particle sizer/nm	153	182	206	227	235

**Table 2:** Effect of the PU/PA ratio on the properties of the emulsion.

PU/PA	1:1	1:2	1:3	1:4	1:5	1:6	1:7
Emulsion stability	Unstable	Stable	Stable	Stable	Stable	Stable	Stable
Water absorption (%)	14.5	10.1	9.3	7.1	5.7	4.2	4.0
Solvent resistance (THF)	Almost dissolved	Most dissolved	Partially dissolved	Partially dissolved	corrosion	swelling	swelling
Elongation at break (%)	488.1	373.1	287.5	219.4.	190.1	173.2	150.1
Pencil hardness	HB	HB	H	H	H	2H	2H

**Table 3:** Effect of water-soluble monomer addition method on the properties of emulsion.

Addition Method	MAA (Pre-Emulsion)/AA (Pre-Emulsion)	MAA (Pre-Emulsion)/AA (Seed)	MAA (Seed)/AA (Pre-Emulsion)	MAA (Seed)/AA (Seed)
Emulsion stability	Stable	Stable	Stable	Unstable
Viscosity/mPa∙s	133	156	152	144
particle sizer/nm	185	192	210	197
PdI	0.15	0.10	0.12	0.32

**Table 4:** Effect of selection of emulsifiers on the properties of emulsion.

Emulsifier	Viscosity (mPa∙s)	Particle Sizer (nm)	PDI	Storage Stability	Chemical Stability	Film Appearance
SDS/OP-10	327	173.9	0.218	Stable	demulsification and stratification	shrinkage cavity
SE-10	165	184.6	0.172	Stable	No demulsification and stratification	Smooth
